# Psychometric validation of the work productivity and activity impairment questionnaire in ulcerative colitis: results from a systematic literature review

**DOI:** 10.1186/s41687-018-0088-8

**Published:** 2018-12-13

**Authors:** Aaron Yarlas, Stephen M. Maher, Martha S. Bayliss, Andrew Lovley, Joseph C. Cappelleri, Marco D. DiBonaventura

**Affiliations:** 1 Optum, 1301 Atwood Avenue, Suite 311N, Johnston, RI 02919 USA; 2 Pfizer, 445 Eastern Point Road, MS 8260-2502, Groton, CT 06340 USA; 3 Pfizer, 235 42nd Street, New York, NY 10017 USA

**Keywords:** Ulcerative colitis, Inflammatory bowel disease, Work productivity and activity impairment questionnaire, Absenteeism, Presenteeism, Work productivity, Literature review

## Abstract

Patients with ulcerative colitis, a type of inflammatory bowel disease, report negative impacts of disease symptoms on work-related outcomes, including absenteeism and presenteeism. As a way to better understand the impact of this disease and its treatment on work-related outcomes, the current review examines the use of the Work Productivity and Activity Impairment Questionnaire (WPAI), a patient-reported outcomes measure of absenteeism, presenteeism, and impairment in other activities, in studies of patients with ulcerative colitis. This review assesses the measurement properties of the WPAI in this patient population: its reliability, construct validity, ability to detect change, and responsiveness to effective treatments. Relevant data were extracted from 13 sources (journal articles and conference posters) identified following a systematic review of the published and gray literature. The evidence supports the WPAI as having test-retest reliability (reproducibility) over time; convergent validity, as indicated by moderate correlations with measures of quality of life and moderate-to-strong correlations with measures of disease activity; known-groups validity, as indicated by differences in WPAI scores between patients with active and inactive disease; ability (sensitivity) to detect change, as indicated by substantial improvement in scores for patients who achieve remission, accompanied by substantial worsening of scores for patients who relapse; and, responsiveness to treatment, with improvements in scores following treatments that reduce disease activity. Limitations included a lack of available evidence from randomized-controlled trials that could speak more directly to the WPAI’s responsiveness to treatment. In conclusion, we recommend the use of the WPAI for measuring work outcomes in both observational studies and interventional trials that include patients with ulcerative colitis.

**Electronic supplementary material:**

The online version of this article (10.1186/s41687-018-0088-8) contains supplementary material, which is available to authorized users.

## Introduction

Ulcerative colitis (UC) is an inflammatory disease of the colon that is characterized by intermittent periods of disease flaring and remission and affects 900,000 patients in the United States alone [1, 2]. Patients with UC experience recurring clinical signs and symptoms, including rectal bleeding, abdominal pain, frequent diarrhea, and an urgent need to defecate. These symptoms are typically assessed in clinical trials using a disease activity index, such as the Mayo score [3] or the Simple Clinical Colitis Activity Index (SCCAI), [4] among others.

What these disease activity measures often fail to capture, however, is the impact of these clinical signs and symptoms on the everyday functioning of patients with UC. One aspect of functioning likely to be affected by recurrent symptoms is work-related outcomes (WRO), such as absenteeism and impairment in productivity at work (i.e., presenteeism). Several studies have used individual or group interviews of patients with inflammatory bowel disease (IBD; inclusive of both UC and Crohn’s disease) to elicit patients’ input on the effect of the disease on patients’ everyday lives, including work experiences [5–10]. Patients in these studies discussed missing work because of disease-related pain, fatigue, or other symptoms [5, 6, 8–10].

Further, in all of these studies, patients described the negative impact on their work performance due to these symptoms, including the difficulty of accessing a toilet and avoiding meetings or interactions with colleagues to hide their symptoms and frequency of toilet use. The increase of absenteeism and presenteeism has been shown to limit employment opportunities for patients with UC; several studies have reported rates of unemployment and use of disability benefits for patients with IBD that were typically two-to-three times higher than those for matched general population controls [11–14]. Further examination of unemployment and disability rates found that these differences disappear when only including IBD patients who are asymptomatic or in remission [15, 16].

Collectively, these results indicate that patients with active UC have impaired WRO and that inducing remission may improve these outcomes. Thus, it is important that clinical trials capture the degree to which the studied treatment may be able to improve WRO. Since objective data on WRO (e.g., absentee information from employment databases) are difficult to obtain in a clinical trial setting, self-reported measures are typically used to assess the impact of disease and treatment on absenteeism and presenteeism. Several patient-reported outcomes measures (PROMs) have been developed for this purpose; among the most frequently used is the Work Productivity and Activity Impairment questionnaire (WPAI) [17].

The WPAI measures the impact of health problems on absenteeism, presenteeism, overall work performance, and non-work activities. The WPAI has been shown to be reliable, valid, and responsive when used with patients across several disease areas, including those with gastrointestinal conditions (e.g., irritable bowel syndrome, [18, 19] gastroesophageal reflux disease [20], and Crohn’s disease [21, 22]). However, there has not (to our knowledge) yet been an examination of the measurement properties of the WPAI when used with samples of patients with UC; as such, we believe that this article is the first to do so.

The objective of this paper is to report results from the first systematic literature review on the measurement properties of the WPAI when used with UC patients. Studies included in this review were identified within both the published and unpublished (or “gray”) literature. Evidence was synthesized across identified studies for the purpose of examining evidence for the instrument’s reliability, construct validity, ability to detect change, and responsiveness to treatment for the UC patient population.

## Methods

### WPAI

The WPAI (presented in Additional file 1) is a self-administered six-item survey designed to measure the impact of a person’s health problems on WRO over the previous seven days [17]. This includes work time missed (absenteeism), impaired productivity at work (presenteeism), overall work impairment (OWI; combined absenteeism and presenteeism), and impairment in non-work-related activities due to health problems (activity impairment), over the previous seven days.

Depending on how questions are framed, the WPAI can measure the impact of general health problems (WPAI-GH), or the impact of a specific health problem. In the latter case, the name of the condition is usually included; for example, if subjects are asked to answer the questions regarding the impact of UC specifically (rather than “health problems” generically, as in the WPAI-GH), the instrument would be referred to as the WPAI-UC.

### Systematic literature review

#### Search sources and terms

The literature search and selection process adhered to guidelines described in the Preferred Reporting Items for Systematic Reviews and Meta-Analyses (PRISMA) Statement [23]. In particular, we conducted searches of PubMed, Embase, and the Cochrane Register of Controlled Trials (CENTRAL) for publications in peer-reviewed journals. We also searched for posters and presentations at peer-reviewed national and international conferences on topics of gastrointestinal diseases and measurement of patient-reported outcomes (PRO) using Embase and the International Society for Pharmacoeconomics and Outcomes Research (ISPOR) scientific presentations database. All searches were performed in November 2017. Search terms were designed to capture studies in which the WPAI was administered to patients with UC or IBD more generally (see Additional file 1 for full search strings).

In addition, we used the search engine on the ClinicalTrials.gov website, using the keywords “work productivity” and “WPAI” with conditions of “inflammatory bowel disease” and “ulcerative colitis.” We also reviewed references listed on the WPAI developer’s webpage (http://www.reillyassociates.net/WPAI_References.html) and those cited in selected records. The protocol outlining the search strategy is available upon request from the authors.

#### Selection of records (articles and posters)

Screening of each record at each stage of review was conducted by at least two of three independent reviewers (AY, SM, and AL). Discrepancies at any stage of review were discussed by all three reviewers until consensus was reached.

Initial screening was based on articles’ titles and abstracts. Full-text articles/posters of records not excluded during title/abstract screening were retrieved to perform a further review. Records for which the full-text was available online or for purchase were retrieved directly. For items not directly available via these means, authors were contacted via email (and phone, when possible) in an attempt to retrieve the full text.

At each screening phase, records were selected if they met (or, during abstract screening, did not clearly fail to meet) the following inclusion criteria: published in English, and provided quantitative WPAI-GH or WPAI-UC data for adult patients with UC (or within a sample of IBD patients, with data reported separately for a UC patient subgroup) that could be used to assess the instrument’s reliability, validity, responsiveness, or sensitivity to treatment. Data reported numerically were extracted directly from selected records and added to a database to be summarized. Data reported only graphically (i.e., in a figure) were extracted using WebPlotDigitizer-Desktop, version 2.8 (https://automeris.io/WebPlotDigitizer), a computer program that uses the spatial distances of the axes to determine the numeric values provided.

### Assessment of measurement properties of the WPAI in UC

#### Reliability

Given that each of the single WPAI items captures a distinct and independent construct, internal consistency was not considered for review as it requires multiple items per construct. Test-retest reliability, or reproducibility, of WPAI domains was evaluated from studies assessing the magnitude of change in WPAI domain mean scores across two time points for patients with stable disease activity during the interval (e.g., were not in remission at either time, or were in remission at both times). Evidence that changes in WPAI domains were small, and do not exceed established clinically important change (CIC) thresholds, would support the instrument as having adequate test-retest reliability.

#### Construct validity

Construct validity was assessed through both convergent validity and known-groups validity. Convergent validity assesses whether instruments that purport to measure the same construct (or conceptually similar constructs) show strong concordance. Convergent validity was examined by evaluating correlations between WPAI domain scores and scores from instruments measuring conceptually related constructs, including health-related quality of life (HRQoL) and UC disease activity. Evidence for acceptable convergent validity were correlation coefficient values ≥|0.40|, with correlations <|0.40| but ≥|0.30| not considered as evidence to dismiss convergent validity, as recommended for determination of this property when using PRO measures [24]. Known-groups validity assesses whether the scores from the instrument differ across groups known to differ on that construct (or conceptually similar construct). Known-groups validity was assessed by examining the magnitude of differences in WPAI domain scores between groups that were known to differ in UC disease activity (e.g., active disease vs. remission), or specific health-relevant symptoms (e.g., fatigue).

#### Ability to detect change

The ability of the WPAI domains to detect changes in UC disease activity was evaluated by assessing the magnitude of change in WPAI domain mean scores for patients showing clinically meaningful changes in activity status (i.e., a change from active disease to remission, or vice-versa, based on pre-specified criteria from a disease activity index). Evidence that changes in WPAI domains exceed a level that indicates clinical importance would support the instrument as able to detect change. CICs have been conceptually defined as the smallest change in score which patients perceive as beneficial and for which a clinician would recommend a change in the patient’s care [25]. Evaluation of within-patient change in scores for the WPAI-CD (i.e., the WPAI where “health problems” is replaced with “Crohn’s disease”) using both distribution-based methods and anchor-based methods (defined by changes on the CD activity index [CDAI] [26]) found that a change of 7% in each WPAI-CD domain corresponds to a clinically meaningful change in CD patients’ disease activity [21, 27]. Despite the lack of direct evaluation of a CIC for the WPAI-UC, given the similarities between symptoms of the two conditions, previous research on the WPAI in UC patients have adopted these CIC thresholds [28, 29].

#### Responsiveness to treatment

The responsiveness of the WPAI domains to treatments shown to be effective for reducing UC activity was evaluated by assessing the magnitude of change in WPAI domain mean scores from baseline to post-treatment assessment. Evidence that changes in WPAI domains exceed established CIC thresholds would support the instrument as responsive to treatment.

## Results

### Literature review

**Fig. 1:**
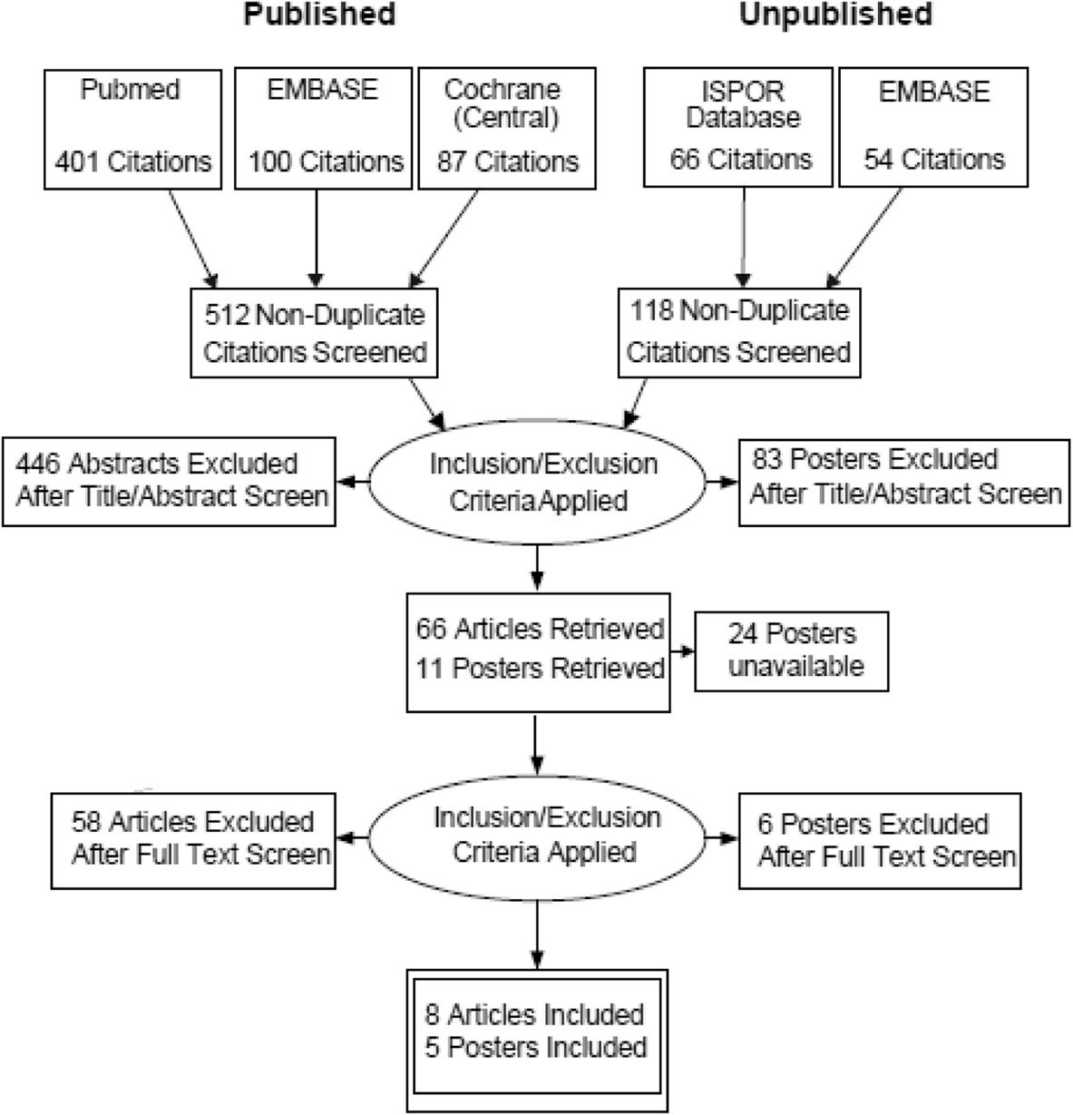
PRISMA flow chart for results of search for published and unpublished literature

The number of records retrieved from each queried source, the number excluded from the review at each stage of selection, and the number selected for review, for both published articles and unpublished conference posters, are reported in the PRISMA flow chart in Fig. 1. Data from 13 records – eight articles [29–36] and five posters [37–41] – that met all selection criteria were identified from the literature search.

We used two tools to assess the quality of these 13 studies. The first tool, the Joanna Briggs Institute (JBI) critical appraisal checklist for analytical cross sectional studies, [42] was used to evaluate the quality of the eight non-interventional cross-sectional studies [30–35, 38, 39] identified from our literature search. The second tool, the National Institute of Health (NIH) quality assessment tool for observational cohort and cross-sectional studies, [43] was used to evaluate the quality of the five open-label non-comparative interventional studies [29, 36, 37, 40, 41] identified in our literature search. Neither of these tools provides a clear-cut algorithm for determining the quality of the study or for deciding whether the study should be included in or excluded from a review; rather, the decision is left to the reviewer based on the pattern of checklist responses.

Based on the patterns of responses to the JBI checklist, each of the eight cross-sectional studies was appraised as having sufficient quality to be rated as “include”, and so data from all of these studies were included in the review. Based on the patterns of responses to the checklist within the NIH quality assessment tool, each of the five open-label non-comparative interventional studies was given a quality rating of either “good” or “fair”, and so data from all of these studies were included in the review. Sample and design characteristics of the 13 reviewed studies are presented in Table 1.

**Table 1:** Sample and study design characteristics of selected records

Study	Article/ poster	Study design	Sample description	Disease status	WPAI version
Cohen 2014 [30]	Article	Cross-sectional	220 patients with IBD (95 with UC) enrolled in a patient registry in the US in 2008–2009	Mixed (75% remission, 25% active)	WPAI:GH
Gibson 2014 [31]	Article	Cross-sectional	175 outpatients with UC attending specialist consultations in Australia in 2011	Mixed (54% remission, 46% active [17% mild, 30% moderate/severe])	WPAI:UC
Mandel 2014 [32]	Article	Cross-sectional	443 patients with IBD (183 with UC) at specialized IBD centers in Hungary	Mixed (75% remission, 25% active)	WPAI:UC
Meijs 2014 [33]	Article	Cross-sectional	58 inpatients with UC that were hospitalized in the Netherlands in 2008–2011	Remission (50% medication, 50% post-surgical)	WPAI:UC
Travis 2017 [29]	Article	Open-label non-comparative interventional	463 patients with UC from 92 study sites in Europe, Asia, and North America in 2012-2015	All active at study baseline (moderate/severe)	WPAI:GH
Vaizey 2014 [34]	Article	Cross-sectional	173 outpatients with UC visiting a gastroenterologist in the UK in 2011–2012	Mixed (58% remission, 42% active [18% mild, 24% moderate/severe])	WPAI:UC
van Assche 2016 [35]	Article	Cross-sectional	253 patients with UC recruited at investigative sites from 11 European countries	Mixed (20% remission, 80% active)	WPAI:UC
Yarlas 2015a [36]	Article	Open-label non-comparative interventional	309 patients with UC recruited at investigative sites from the US in 2007–2009	All active at acute phase baseline, all in remission at maintenance phase baseline	WPAI:UC
Jackson 2016 [38]	Poster	Cross-sectional	81 patients with IBD (31 with UC) attending a tertiary clinic in Australia in 2015	Mixed (NOS)	WPAI:GH
Katz Avitan 2016 [39]	Poster	Cross-sectional	405 patients with IBD (150 with UC) attending a tertiary hospital in Israel 2015	Mixed (67% remission, 33% active)	WPAI:UC
Willshire 2014 [40]	Poster	Open-label non-comparative interventional	717 patients with UC recruited at investigative sites globally	All active at acute phase baseline, all in remission at maintenance phase baseline	WPAI:UC
Yarlas 2014 [41]	Poster	Open-label non-comparative interventional	717 patients with UC recruited at investigative sites globally	All active at acute phase baseline, all in remission at maintenance phase baseline	WPAI:UC
Yarlas 2015b [37]	Poster	Open-label non-comparative interventional	717 patients with UC recruited at investigative sites globally	All active at acute phase baseline, all in remission at maintenance phase baseline	WPAI:UC

*GH* general health, *IBD* inflammatory bowel disease, *NOS* not otherwise specified *UC* ulcerative colitis, *UK* United Kingdom, *US* United States, *WPAI* Work Productivity and Activity Impairment questionnaire

At least one of the, and often several, authors of the 24 unavailable posters were contacted to obtain the posters. From these contacts, replies were received from three authors who declined to share results: one claimed their data were proprietary content, one stated that the data were already published (these published data have been included in this review [29]), and one stated that the data were qualitative in nature. For the remaining posters, no authors responded to our requests after sending three separate emails.

### Reliability

Test-retest reliability of WPAI domains was evaluated based on results from one study [37] that compared scores at the start and end of an open-label maintenance treatment phase period for patients whose remission status (partial remission or complete remission) was unchanged, as determined by scores on the Ulcerative Colitis – Disease Activity Index (UC-DAI) [44]. As reported in Table 2, differences in percentages, even across 12 months, were less than 5% for each domain, none of which exceeded the CIC threshold of 7% for clinically meaningful change. No measure of association (e.g., intraclass correlation coefficient) was reported for these data.

**Table 2:** Changes in WPAI scores over time for patients with no change in UC disease activity status

				Change in mean WPAI scores from Time 1 to Time 2			
Study	N	Status at both assessments	Interval	Absenteeism	Presenteeism	OWI	Activity Impairment
Yarlas 2015b [37]	45	Complete remission^a^	12 months^c^	− 0.5	−4.2	− 4.8	0.2
Yarlas 2015b [37]	53	Partial remission^b^	12 months^c^	− 4.3	−1.1	−4.9	− 1.6
Weighted Mean differences^d^				− 2.6	−2.5	−4.9	−0.8

*OWI* Overall Work Impairment, *UC* ulcerative colitis, *WPAI* Work Productivity and Activity Impairment questionnaire

^a^Complete remission defined as UC-DAI total score ≤ 1, rectal bleeding and stool frequency components = 0, and ≥ 1-point reduction in endoscopy score from induction phase baseline

^b^Partial remission defined as UC-DAI total score ≤ 3, combined score on rectal bleeding and stool frequency components ≤1, and not in complete remission

^c^Receiving multimatrix mesalamine 2.4 g/day once daily

^d^Weighted by sample size

### Convergent validity

#### Correlations with criterion measures of HRQoL

Evidence for convergence between WPAI domains and measures of HRQoL was evaluated based on results from one study of patients with UC [36]. This study reported Spearman rank-order correlation coefficients between WPAI domains and subscales from two HRQoL measures: the Short Inflammatory Bowel Disease Questionnaire (SIBDQ), [45] and the SF-12® Health Survey, version 2 (SF-12v2) [46]. Both of these measures were deemed as appropriate for testing the convergent validity of the WPAI because their parent instruments (the SF-36 and the IBDQ, respectively) had been included as criteria for testing the convergent validity of the WPAI-CD [21]. Because higher subscale scores for these two measures indicate better HRQoL, and higher scores on WPAI indicate compromised work productivity or activity impairment, it was expected that all correlation coefficients would be in the negative direction.

As reported in Table 3, all correlations between the domains of the WPAI and the SIBDQ were negative in direction, as expected, ranging from − 0.13 to − 0.68 (overall median = − 0.47). All WPAI domains met criteria for convergence (≥0.40) [24] with the SIBDQ Bowel symptoms subscale. All WPAI domains, with the exception of absenteeism, were convergent with the SIBDQ Social function subscale. Presenteeism and activity impairment domains were convergent with the SIBDQ Emotional function subscale, while only the activity impairment domain was convergent with the SIBDQ Systemic symptoms subscale. Across SIBDQ subscales, convergence was supported for presenteeism, OWI, and activity impairment (median correlations ranging from − 0.51 to − 0.52).

**Table 3:** Convergent validity: Spearman rank-order correlation coefficients between WPAI domains and criterion measures of health-related quality of life

	WPAI Domain			
Criterion Measure	Absenteeism	Presenteeism	Overall Work Impairment	Activity Impairment
SIBDQ				
*Bowel symptoms*	**−0.47**	**−0.68**	**− 0.68**	**−0.55**
*Systemic symptoms*	−0.13	−0.31	− 0.31	**−0.46**
*Emotional function*	−0.15	**−0.40**	− 0.38	**−0.49**
*Social function*	−0.36	**−0.64**	**− 0.63**	**−0.68**
*Median correlation*	*−0.26*	***−0.52***	***− 0.51***	***−0.52***
SF-12v2				
*Physical functioning*	−0.22	− 0.28	− 0.28	−0.35
*Role physical*	−0.38	**−0.57**	**− 0.57**	**−0.50**
*Bodily pain*	**−0.52**	**−0.54**	**− 0.54**	**−0.55**
*General health*	−0.07	−0.15	− 0.12	−0.34
*Vitality*	−0.33	−0.31	− 0.31	**−0.44**
*Social functioning*	−0.37	−0.35	− 0.36	**−0.44**
*Role emotional*	−0.35	**−0.46**	**− 0.46**	**−0.41**
*Mental health*	−0.18	−0.17	− 0.17	−0.33
*Median correlation*	*−0.34*	*−0.33*	*− 0.34*	***−0.43***

*SF-12v2* SF-12v2 Health Survey, *SIBDQ* Short Inflammatory Bowel Disease Questionnaire, *WPAI* Work Productivity and Activity Impairment questionnaire

Note: all data in this table are from Yarlas et al (2015a) [36]

Values printed in **BOLD** indicate correlations satisfying the definition of convergent validity (≥|0.40|) [24]

All inter-scale correlations between WPAI domains and SF-12v2 subscale scores were in a negative direction and ranged from − 0.07 to − 0.57 (overall median = − 0.35). The WPAI domains showed the greatest degree of convergence with the Bodily pain subscale (all domains meeting criteria), followed by the Role physical and Role emotional subscales (presenteeism, OWI, and activity impairment domains met criteria for both). These associations with SF-12v2 Role limitations subscales would be expected, as they assess similar constructs as the WPAI domains, namely the impact of health problems on patients’ work productivity and ability to engage in other activities [46]. Across SF-12v2 subscale scores, convergence was supported only for the activity impairment domain (median correlation of − 0.43), though median correlations for the other three domains (ranging from − 0.33 to − 0.34) were large enough that convergent validity cannot be dismissed.

#### Correlations with criterion measures of disease activity

Evidence for convergence between measures of disease activity and the domains of the WPAI was evaluated based on three studies [32, 38, 41] of UC patients that reported Spearman rank-order correlation coefficients between WPAI domains and scores from one of three measure of disease activity: the partial Mayo score, [3] the SCCAI, [4] and the UC-DAI [44]. Because higher scores for each of these measures indicate increased disease activity, it was expected that all correlation coefficients with WPAI would be positive values.

As reported in Table 4, all correlations were positive. Inter-scale correlations between WPAI domains and disease activity measures ranged from 0.32 to 0.85 (median = 0.45). The OWI domain was convergent with disease activity measures in all three studies, while the presenteeism and activity impairment domains were convergent with disease activity measures in two of the three studies (correlations with the absenteeism domain were only reported in two studies; convergence with disease activity was found in one of these). Across studies, convergence with disease activity was supported for presenteeism, OWI, and activity impairment (median correlations ranging from 0.43 to 0.60), with the median correlation for absenteeism (0.39) large enough to not be considered as evidence to dismiss convergent validity.

**Table 4:** Convergent validity: Correlations between WPAI domains and criterion measures of disease activity

		WPAI Domain			
Study	Criterion Measure	Absenteeism	Presenteeism	OWI	Activity Impairment
Jackson 2016 [38]	SCCAI	–	**0.76**	**0.85**	**0.83**
Mandel 2014 [32]	Partial Mayo score	**0.45**	**0.51**	**0.60**	0.37
Yarlas 2014 [41]	UC-DAI	0.32	0.34	**0.42**	**0.43**
*Median correlation*		*0.39*	***0.51***	***0.60***	***0.43***

*OW*, Overall Work Impairment; *SCCAI* Simple Clinical Colitis Activity Index; *UC-DAI* Ulcerative Colitis – Disease Activity Index; *WPAI* Work Productivity and Activity Impairment questionnaire

-- Value not reported

Values printed in **BOLD** indicate correlations satisfying the definition of convergent validity (≥|0.40|) [24]

### Known-groups validity

Further evidence supporting the associations between WPAI domain scores and other health outcomes in UC patients was examined using the known-groups approach for discriminant validity. Differences in mean WPAI domain scores were calculated between subgroups of patients classified by health status in 11 comparisons from eight studies [30, 31, 34–37, 39, 41].

As reported in Table 5, across all studies, patients in subgroups defined by worse health outcomes (e.g., active disease; presence or lack of improvement in UC symptoms) scored clinically meaningfully worse on all WPAI domains than did patients in corresponding subgroups of better health outcomes. Summarized differences indicate that patients with worse health outcomes scored approximately 20–25% higher (worse) on presenteeism, OWI, and activity impairment domains, and approximately 12% worse on the absenteeism domain, than did patients with better health outcomes. Differences exceeded the CIC threshold of 7% for absenteeism in eight of the 11 comparisons, presenteeism in 10 of the 11 comparisons, and OWI and activity impairment in all comparisons.

**Table 5:** Differences in WPAI scores between UC patient subgroups differing on health status

				Group mean differences in WPAI domain scores			
Study	N	Predictor	Comparison	Absenteeism	Presenteeism	OWI	Activity Impairment
Gibson 2014 [31]	175	Remission status (partial Mayo)	Active vs Remission	**20.3**	**13.4**	**17.2**	**27.8**
Vaizey 2014 [34]	173	Remission status (partial Mayo)	Active vs Remission	**22.8**	**21.2**	**26.4**	**29.7**
Katz Avitan 2016 [39]	150	Remission status (SCCAI)	Active vs Remission	**24.5**	**36.7**	**42.1**	–
van Assche 2015 [35]	242	Remission status (self-perceived)	Active vs Remission	**10.4**	**22.0**	**23.0**	**24.0**
Yarlas 2015b [37]	180	Remission status, Month 12 (UC-DAI)	Active vs Remission	**11.7**	**25.6**	**34.3**	**26.5**
Yarlas 2015b [37]	258	Remission status, Week 8 (UC-DAI)	Active vs Remission	**7.4**	**22.6**	**26.7**	**26.0**
Yarlas 2014 [41]	180	Remission status (UC-DAI)	Active vs Remission	5.0	5.3	**11.1**	**9.7**
Yarlas 2014 [41]	180	Improved rectal bleeding (UC-DAI)	Not improved vs improved	6.3	**7.8**	**12.2**	**12.3**
Yarlas 2014 [41]	180	Improved stool frequency (UC-DAI)	Not improved vs improved	**12.5**	**13.8**	**20.0**	**19.2**
Yarlas 2015a [36]	146	Clinical symptom recurrence (UC-DAI)	Recurrent vs Non-recurrent	−0.9	**12.7**	**12.5**	**9.2**
Cohen 2014 [30]	95	Fatigue (FACIT-Fatigue)	Fatigue vs Non-fatigue	**11.3**	**23.9**	**23.8**	**25.7**
Weighted mean difference^a^				***11.7***	***18.5***	***22.7***	***21.3***
Median difference				***11.3***	***21.2***	***23.0***	***24.9***
Percentage of values exceeding CIC				*73%*	*91%*	*100%*	*100%*

*CIC* clinically important change; *FACIT* Functional Assessment of Chronic Illness Therapy; *OWI* Overall Work Impairment; *SCCAI* Simple Clinical Colitis Activity Index; UC, ulcerative colitis; *UC-DAI* Ulcerative Colitis – Disease Activity Index; *WPAI* Work Productivity and Activity Impairment questionnaire

^a^Weighted by sample size

--Reported value were not within the valid range (i.e., was > 100)

Values printed in **BOLD** indicate mean differences exceeding the CIC threshold of 7% [27]

### Ability to detect change

The ability of WPAI domains to detect changes in underlying UC disease activity was evaluated by assessing the magnitude of change in WPAI domain mean scores for patients showing clinically meaningful changes in activity status (i.e., a change from active disease to remission, or vice-versa). One study compared assessments of mean WPAI domain scores from an open-label treatment study for two groups of patients: 1) patients with active UC who achieved remission following eight weeks of once-daily treatment with multimatrix mesalamine 4.8 g/day and 2) patients with UC in remission who relapsed after 12 months in an extension phase during which they received once-daily treatment with multimatrix mesalamine 2.4 g/day once daily for 12 months [37].

As reported in the top row of Table 6, patients with active disease who achieved remission at Week 8 reported an approximately 25–30% decrease (i.e., improvement) in presenteeism, OWI, and activity impairment and an approximately 9% decrease in absenteeism, all exceeding thresholds indicating clinically meaningful change. On the other hand, patients in the extension phase who relapsed at Month 12 reported an approximately 20–25% increase in presenteeism, OWI, and activity impairment, and an approximately 9% increase in absenteeism, all exceeding thresholds indicating clinically meaningful change. These results indicate the ability of WPAI domains to detect both positive change and negative change in patients’ UC activity.

**Table 6:** Changes in WPAI scores over time as a function of change in UC disease activity status

					Change in mean WPAI scores from Time 1 to Time 2			
Study	N	Time 1 status	Time 2 status	Interval	Absenteeism	Presenteeism	OWI	Activity Impairment
Yarlas 2015b [37]	180	Active	Remission	8 weeks^a^	**− 8.7**	**−26.0**	**− 28.0**	**−30.1**
Yarlas 2015b [37]	258	Remission	Active	12 months^b^	**9.4**	**19.4**	**25.4**	**22.3**

*OWI* Overall Work Impairment; *UC* ulcerative colitis; *WPAI* Work Productivity and Activity Impairment questionnaire

^a^Receiving multimatrix mesalamine 4.8 g/day once daily

^b^Receiving multimatrix mesalamine 2.4 g/day once daily

Remission defined as UC-DAI total score ≤ 1, rectal bleeding and stool frequency components = 0, and ≥ 1-point reduction in endoscopy score

from induction phase baseline

Values printed in **BOLD** indicating those exceeding established thresholds indicating clinically important change

### Responsiveness to treatment

The responsiveness of WPAI domains to effective treatment was evaluated by assessing the magnitude of change in WPAI domain mean scores reported at pre-treatment and post-treatment visits in studies using non-comparative treatment intervention designs. The change in WPAI domain scores over eight weeks from two prospective open-label studies of multimatrix mesalamine treatment (one administering 2.4–4.8 g/day once daily, [36] the other 4.8 g/day once daily [40]) and one prospective open-label study of adalimumab treatment (160/80 mg at Weeks 0/2 followed by 40 mg every other week through Week 26) [29] was evaluated. Results based on primary efficacy analyses of each study showed that treatment was effective in inducing clinical response and remission [29, 47].

As reported in Table 7, summaries of change following treatment across these three studies indicate that patients reported an approximately 20% decrease in presenteeism, OWI, and activity impairment and an 8% decrease in absenteeism, all exceeding thresholds indicating clinically meaningful change.

**Table 7:** Responsiveness to treatment: change in WPAI scores following effective treatment interventions in prospective, open-label studies

				Change (mean WPAI scores at Week 8 - mean WPAI scores at baseline)			
Study	N	Treatment regimen	Treatment duration	Absenteeism	Presenteeism	OWI	Activity Impairment
Travis 2017 [29]	446	ADA (initial 160/80 mg, 40 mg EOW at Week 4)	26 weeks	**−11.4**	**−24.5**	**− 29.2**	**−27.2**
Willshire 2014 [40]	404	MMX mesalamine 4.8 g/day	8 weeks	**− 7.6**	**−20.6**	**−23.4**	**−24.1**
Yarlas 2015a [36]	103	MMX mesalamine 2.4–4.8 g/day	8 weeks	− 4.0	**−12.3**	**− 13.8**	**−14.2**
Weighted mean change^a^				***− 9.0***	***−21.5***	***−25.1***	***−24.5***
Median change				***−7.6***	***− 20.6***	***−23.4***	***−24.1***

*ADA* adalimumab, *EOW* every other week, *MMX* multimatrix, *OWI* Overall Work Impairment, *WPAI* Work Productivity and Activity Impairment questionnaire

Values printed in **BOLD** indicating those exceeding established thresholds indicating clinically important change

^a^Weighted by sample size

## Discussion

This first review (to our knowledge) of the measurement properties of the WPAI when used with UC patients shows the instrument to be reliable, valid, able to detect change, and responsive to treatment when used to assess WRO in this patient population. Results from reviewed studies found evidence that WPAI domain scores were reproducible after 12 months in patients who showed no change in underlying disease condition, and showed change in the expected direction among patients whose disease status improved (e.g., achieved remission) or worsened (e.g., relapsed) over time.

Two kinds of evidence supported the validity of the WPAI. First, convergent validity was supported by findings of higher scores on WPAI domains, particularly presenteeism, OWI, and activity impairment, being associated with lower scores on measures of HRQoL and with higher scores on indices of disease activity – that is, worse scores on WPAI domains were associated with worse scores on health status and disease activity, as expected. Second, known-groups validity was supported by findings that WPAI scores were substantially higher for patients with worse disease activity and more severe symptoms, again as expected. Divergent validity was not examined in this review, due to a lack of relevant data from identified sources.

Ability to detect change was supported by evidence of decreases in WPAI scores for patients with improved disease status (i.e., patients in active disease who achieved remission) and by increases in WPAI scores for patients with worsened disease status (i.e., patients in remission who relapsed to active disease). Responsiveness to treatment was evidenced by substantial decreases in scores for patients who received effective treatments. The magnitude of differences in scores over time and by condition exceeded established CIC thresholds, indicating that changes and differences were clinically meaningful.

Measurement properties were weaker for the WPAI absenteeism domain relative to the other three domains. This is likely related to the highly skewed distribution observed for the absenteeism domain in most studies, with the majority of responses indicating zero days absent in the past seven days due to health problems. For example, in Yarlas et al. (2015a) [36], a response of zero was observed in 73% of subjects with active UC (as compared to approximately 25% in the other three domains), meaning that positive change (reduction) in the absenteeism domain was only possible for one-quarter of subjects. This limits the magnitude of improvements that can be observed as a function of treatment or accompanying change in disease status, and the restricted range also can lead to underestimation of correlations with other variables [48].

The magnitudes of association for WPAI domains with criterion HRQoL measures varied across the domains of those measures. Specifically, all WPAI domains showed stronger associations with the SIBDQ Bowel symptoms and Social function domains than with the Systemic symptoms and Emotional function domains. Most WPAI domains also correlated more strongly with the SF-12v2 Bodily pain, Role physical, and Role emotional domains than other domains of the SF-12v2. These patterns of associations are logical from both a clinical and content perspective. From a clinical perspective, bowel symptoms, such as stool urgency and abdominal pain, would lead to impairment in work and activity impairment more than mental health or perception of general health. From a content standpoint, the SIBDQ Social function domain and the SF-12v2 Role physical and Role emotional domains assess constructs involving the impact of health on work and other activities, and thus would be expected to strongly correlate with WPAI domains. So, while divergent validity was not explicitly examined in this review, the patterns of magnitudes of convergence with HRQoL domains are consistent with content overlap.

The majority of studies included in this review reported administering the UC-specific version of the WPAI (i.e., WPAI-UC), with only three studies [29, 30, 38] reporting use of the general health version (i.e., WPAI-GH). One would expect more precision in findings from studies using the WPAI-UC than the WPAI-GH, since the former is more specific to UC activity, but in fact this was not observed in our review, as findings were comparable across both types of instruments. For studies using the WPAI-GH, this may be indicative of the fact that UC accounted for the majority of health problems in these patients, or that within the context of these studies, where the focus was on patients’ UC, patients responses were driven by their perceptions of their UC-specific health even without explicit instruction. However, to maximize the sensitivity of the instrument, we recommend that the disease-specific version be used as a trial endpoint.

There are some limitations in the current review and gaps in the extant literature that require discussion. First, the evidence base was rather small. Evidence for test-retest reliability, ability to detect change, and convergence with HRQoL measures were each based on findings from a single study, while evidence for convergence with measures of disease activity and responsiveness to treatment were based on only three studies each.

The evidence base was limited due the lack of potentially relevant data. We conducted a systematic, comprehensive search of both the published and unpublished literature. However, our search of the unpublished ‘gray’ literature was focused on presentations and posters at conferences deemed relevant to the topic and accessible through the Embase database (as well as the ISPOR conference). Further, as discussed earlier, there were two dozen conference presentations that we were unable to retrieve, either due to the authors’ refusal to provide or our inability to get a response from authors even after repeated contact attempts. Assuming that the availability of presentations was unrelated to the findings reported (which was supported by the titles and abstracts of unavailable presentations), then the findings presented here should be unbiased and generalizable.

Another point for consideration is the quality of the studies included in this review. While all 13 identified studies were judged as acceptable for inclusion during our quality assessment process, most of these studies, or at least their description of them, had weaknesses identified during this process. Most of the posters reported only cursory descriptions of the study sample, limiting the ability to generalize their findings to a larger patient population. None of the studies provided any justification for sample size; however, since we do not consider statistical significance of findings in our review, but rather effect sizes or magnitudes of differences/change, this limitation is not too relevant to our findings.

All but one of the 13 studies failed to make statistical adjustments or use another strategy to identify or control for confounding factors in their statistical models, which was an item on both the JBI and NIH checklists. This failure to control for possible confounding factors means that the effect sizes and magnitude of differences and change in our assessments of validity, ability to detect change, and responsiveness may be biased. Findings from this review of evidence in the literature is bound by the quality of that evidence. While no study was judged to be of poor enough quality that required exclusion from the review, all reviewed studies were flawed in some way, and so the possibility of bias or limits to the generalizability of our findings cannot be fully dismissed.

We examined the responsiveness of the WPAI to effective treatment in observational studies, but did not identify publications of randomized-controlled trials (RCTs) with UC patients in which the WPAI was included as an endpoint. This limited our ability to examine its responsiveness (within-patient change) and sensitivity to detect treatment differences (between-group difference) in RCTs. We are aware of recent, unpublished RCTs with UC patients in which the WPAI was administered, and hope that results from these studies will be made available soon so that the instrument can be evaluated for this purpose.

Another important gap in the existing literature is that no identified studies have solely examined the measurement properties of the WPAI. The review here relied on use of the WPAI data in studies that had other research purposes. A study dedicated to examining the psychometric profile of the WPAI in a large UC sample would provide a much needed base of information regarding these properties.

## Conclusion

This review found that the WPAI has demonstrated good measurement properties in studies of UC patients. Findings from these studies included evidence that WPAI domains have adequate test-retest reliability; convergent validity with measures of HRQoL and UC disease activity; discriminant validity in predicting classification of patients by UC disease activity or other health-related outcomes; ability to detect changes in disease activity; and evidence of responsiveness to effective treatment. At the same time, because of the limited evidence base in the published and unpublished literature, these findings should be considered encouraging though preliminary until more evidence emerges.

## Additional file

Additional file 1: Appendix 1. Work Productivity and Activity Impairment Questionnaire (WPAI). Appendix 2. Terms and Strings Used in Electronic Database Literature Searches. (DOCX 21 kb)

## Abbreviations

CDAI: Crohn’s Disease Activity Index
CENTRAL: Cochrane Register of Controlled Trials
CIC: Clinically Important Change
HRQoL: Health-related Quality of Life
IBD: Inflammatory Bowel Disease
ISPOR: International Society for Pharmacoeconomics and Outcomes Research
OWI: Overall Work Impairment
PRISMA: Preferred Reporting Items for Systematic Reviews and Meta-Analyses
RCT: Randomized Controlled Trial
SCCAI: Simple Clinical Colitis Activity Index
SF-12v2: SF-12v2® Health Survey
SIBDQ: Short Inflammatory Bowel Disease Questionnaire
UC: Ulcerative Colitis
UC-DAI: Ulcerative Colitis Disease Activity Index
WPAI: Work Productivity and Activity Impairment
WPAI:UC: Work Productivity and Activity Impairment, Ulcerative Colitis-Specific Version
WPAI-CD: Work Productivity and Activity Impairment, Crohn’s Disease-Specific Version
WPAI-GH: Work Productivity and Activity Impairment, General Health Version
WRO: Work-Related Outcomes
