# Supplementary material for: Psychometric validation of the work productivity and activity impairment questionnaire in ulcerative colitis: results from a systematic literature review
Source: J Patient Rep Outcomes. 2018 Dec 13;2:62. doi: 10.1186/s41687-018-0088-8 (PMC6292832; doi:10.1186/s41687-018-0088-8)
Supplement: Supplementary file 1 — Appendix 1. Work Productivity and Activity Impairment Questionnaire (WPAI). Appendix 2. Terms and Strings Used in Electronic Database Literature Searches. (DOCX 21 kb) [file 41687_2018_88_MOESM1_ESM.docx]

Appendix 1. Work Productivity and Activity Impairment Questionnaire (WPAI)

**Work Productivity and Activity Impairment Questionnaire:**

**General Health V2.0 (WPAI:GH)**

The following questions ask about the effect of your health problems on your ability to work and perform regular activities. By health problems we mean any physical or emotional problem or symptom. *Please fill in the blanks or circle a number, as indicated.*

1. Are you currently employed (working for pay)? ____ NO ____ YES

*If NO, check “NO” and skip to question 6.*

The next questions are about the **past seven days**, not including today.

2. During the past seven days, how many hours did you miss from work because of your health problems? *Include hours you missed on sick days, times you went in late, left early, etc., because of your health problems. Do not include time you missed to participate in this study.*

*_____*HOURS

3. During the past seven days, how many hours did you miss from work because of any other reason, such as vacation, holidays, time off to participate in this study?

_____HOURS

4. During the past seven days, how many hours did you actually work?

_____HOURS *(If “0”, skip to question 6.)*

5. During the past seven days, how much did your health problems affect your productivity while you were working?

*Think about days you were limited in the amount or kind of work you could do, days you accomplished less than you would like, or days you could not do your work as carefully as usual. If health problems affected your work only a little, choose a low number. Choose a high number if health problems affected your work a great deal.*

Consider only how much health problems affected
productivity while you were working.

| Health problems had no effect on my work |  |  |  |  |  |  |  |  |  |  |  | Health problems completely prevented me from working |
| --- | --- | --- | --- | --- | --- | --- | --- | --- | --- | --- | --- | --- |
|  | 0 | 1 | 2 | 3 | 4 | 5 | 6 | 7 | 8 | 9 | 10 |  |

CIRCLE A NUMBER

6. During the past seven days, how much did your health problems affect your ability to do your regular daily activities, other than work at a job?

*By regular activities, we mean the usual activities you do, such as work around the house, shopping, childcare, exercising, studying, etc. Think about times you were limited in the amount or kind of activities you could do and times you accomplished less than you would like. If health problems affected your activities only a little, choose a low number. Choose a high number if health problems affected your activities a great deal.*

Consider only how much health problems affected your ability
to do your regular daily activities, other than work at a job.

| Health problems had no effect on my daily activities |  |  |  |  |  |  |  |  |  |  |  | Health problems completely prevented me from doing my daily activities |
| --- | --- | --- | --- | --- | --- | --- | --- | --- | --- | --- | --- | --- |
|  | 0 | 1 | 2 | 3 | 4 | 5 | 6 | 7 | 8 | 9 | 10 |  |

CIRCLE A NUMBER

Available from <http://www.reillyassociates.net/WPAI_GH.html>

Note: The WPAI-UC is the same as the WPAI-GH, except “health problems” is replaced by “ulcerative colitis”.

Appendix 2. Terms and Strings Used in Electronic Database Literature Searches

PubMed

Terms appended by [TIAB] were searched only within the title and abstract of articles. Terms appended by [MeSH Terms] were searched only within medical subject headings.

#1 “work productivity and activity impairment” ***OR*** "WPAI" [all fields]

#2 “ulcerative colitis”[TIAB] ***OR*** (colitis, ulcerative)[MeSH Terms] ***OR*** “inflammatory bowel disease”[TIAB] ***OR*** (inflammatory bowel diseases)[MeSH Terms]

#3 English[Language]

#4 #1 ***AND*** #2 ***AND*** #3

Embase

Terms appended by [ab,ti] were searched only within the title and abstract of articles. Terms appended by [lim] limit the search to specified languages.

#1          ‘work productivity and activity impairment’ ***OR*** ‘WPAI’ [all fields]

#2          ‘ulcerative colitis’:ab,ti ***OR*** ‘inflammatory bowel disease’:ab,ti

#3          #1 ***AND*** #2

#4          #1 *AND* #2 [english]/lim

#5 #4

CENTRAL

Terms appended by [ti, ab, kw] were searched only within the title, abstract, and list of keywords of articles, respectively. Terms appended by [MeSH Terms] were searched only within medical subject headings.

#1 “work productivity and activity impairment” *OR* "WPAI" [all fields]

#2 “ulcerative colitis”[ti, ab, kw]

#3 “colitis, ulcerative”[MeSH Terms]

#4 “inflammatory bowel disease”[ti, ab, kw]

#5 “inflammatory bowel disease”[MeSH Terms]

#6 #2 *OR* #3 *OR* #4 *OR* #5

#7 #1 *AND* #6

ISPOR Scientific Presentations Database

#1 Disease/disorder: “GI disorders”

#2 Keyword: “work productivity” in Abstract
